# Online Advertising as a Public Health and Recruitment Tool: Comparison of Different Media Campaigns to Increase Demand for Smoking Cessation Interventions

**DOI:** 10.2196/jmir.1001

**Published:** 2008-12-15

**Authors:** Amanda L Graham, Pat Milner, Jessie E Saul, Lillian Pfaff

**Affiliations:** ^4^New Jersey Department of Health and Senior ServicesTrentonNJUSA; ^3^ClearWay MinnesotaMinneapolisMNUSA; ^2^Healthways QuitNet LLCBostonMAUSA; ^1^Georgetown University Medical Center / Lombardi Comprehensive Cancer CenterWashingtonDCWashingtonDCUSA

**Keywords:** Internet, advertising, consumer demand, smoking cessation

## Abstract

**Background:**

To improve the overall impact (reach × efficacy) of cessation treatments and to reduce the population prevalence of smoking, innovative strategies are needed that increase consumer demand for and use of cessation treatments. Given that 12 million people search for smoking cessation information each year, online advertising may represent a cost-efficient approach to reach and recruit online smokers to treatment. Online ads can be implemented in many forms, and surveys consistently show that consumers are receptive. Few studies have examined the potential of online advertising to recruit smokers to cessation treatments.

**Objective:**

The aims of the study were to (1) demonstrate the feasibility of online advertising as a strategy to increase consumer demand for cessation treatments, (2) illustrate the tools that can be used to track and evaluate the impact of online advertising on treatment utilization, and (3) highlight some of the methodological challenges and future directions for researchers.

**Methods:**

An observational design was used to examine the impact of online advertising compared to traditional recruitment approaches (billboards, television and radio ads, outdoor advertising, direct mail, and physician detailing) on several dependent variables: (1) number of individuals who enrolled in Web- or telephone-based cessation treatment, (2) the demographic, smoking, and treatment utilization characteristics of smokers recruited to treatment, and (3) the cost to enroll smokers. Several creative approaches to online ads (banner ads, paid search) were tested on national and local websites and search engines. The comparison group was comprised of individuals who registered for Web-based cessation treatment in response to traditional advertising during the same time period.

**Results:**

A total of 130,214 individuals responded to advertising during the study period: 23,923 (18.4%) responded to traditional recruitment approaches and 106,291 (81.6%) to online ads. Of those who clicked on an online ad, 9655 (9.1%) registered for cessation treatment: 6.8% (n = 7268) for Web only, 1.1% (n = 1119) for phone only, and 1.2% (n = 1268) for Web and phone. Compared to traditional recruitment approaches, online ads recruited a higher percentage of males, young adults, racial/ethnic minorities, those with a high school education or less, and dependent smokers. Cost-effectiveness analyses compare favorably to traditional recruitment strategies, with costs as low as US $5-$8 per enrolled smoker.

**Conclusions:**

Developing and evaluating new ways to increase consumer demand for evidence-based cessation services is critical to cost-efficiently reduce population smoking prevalence. Results suggest that online advertising is a promising approach to recruit smokers to Web- and telephone-based cessation interventions. The enrollment rate of 9.1% exceeds most studies of traditional recruitment approaches. The powerful targeting capabilities of online advertising present new opportunities to reach subgroups of smokers who may not respond to other forms of advertising. Online advertising also provides unique evaluation opportunities and challenges to determine rigorously its impact and value.

## Introduction

Less than 5% of smokers are able to quit on their own [1,2]. Despite the availability of effective smoking cessation treatments [3], only one in five smokers use proven cessation aids when attempting to quit [4-8]. The population impact (reach × efficacy [9,10]) of cessation treatments could be dramatically increased by increasing the reach and utilization of evidence-based interventions such as telephone- and Web-based programs [3,11,12, 13-16]. Recent expert panels have called for innovative initiatives to increase consumer demand for cessation treatments [17,18]. The National Institutes of Health State-of-the-Science panel on tobacco called for research to “understand the role of different media in increasing consumer demand for and use of effective, individually oriented tobacco cessation treatments for diverse populations” [17].

Historically, consumer demand for cessation treatments has been largely a function of marketing and promotion via traditional media (ie, television, newspaper, radio). Systematic reviews have shown that traditional mass media approaches are effective in increasing treatment utilization [19] and in promoting tobacco cessation when combined with telephone counseling [19,20]. However, there are a number of limitations to traditional media, including costs that can be prohibitive [21]. Mass media approaches tend to yield the lowest participation rates in community-based cessation trials (compared to telephone and other interpersonal methods), reaching only 2.2% of targeted smokers [22]. There is little flexibility with most traditional media formats since it is difficult and costly to switch approaches (eg, changing messages, altering ad content) once funds are spent. From the smoker’s perspective, access to cessation services is one-step-removed, requiring the smoker to write down contact information or to remember a call to action.

Evaluation of the performance of traditional media is challenging as well. It is difficult to randomize to conditions, to control for “spillover” of media into control markets, or to determine how many people actually viewed a billboard or listened to a radio advertisement (ie, the “true denominator” [23]). For television advertising, gross rating points (GRPs) provide an estimate of the percentage of the target audience reached by an advertisement. Otherwise, evaluation relies on the number of individuals who make an initial call to a cessation program and on responses to questions such as “How did you hear of our program?” which may be affected by errors in recall when multiple media campaigns are running simultaneously.

Online advertising may represent a cost-efficient strategy to increase consumer demand for smoking cessation treatments. Approximately 70% of US adults use the Internet [24], and use has increased steadily since 2000 across race, education, income, age, and rural/urban categories [25,26]. As of 2007, a majority of African Americans (62%) and Latinos (78%) reported using the Internet, as did 55% of individuals living in households with an annual income less than US $30,000 [27]. Approximately 12 million adults search for information on quitting smoking each year [28]. Online advertising can be implemented in many forms (eg, banner ads, text ads, or “paid search”), and surveys consistently show that consumers are receptive [29-31]. Online ads can provide smokers with immediate access to Web-based cessation treatments. Ads can be strategically placed on websites with known demographic profiles (eg, Univisión.com for a Latino audience) and geo-targeted by Internet Protocol (IP). Millions of individuals search the Internet each year for information on quitting smoking [28], and paid search ads allow a cessation program to have visibility at the top of the search results where searchers are likely to see the ad.

Perhaps most importantly, online advertising provides new tools for research evaluation to track and estimate impact as well as a real-world laboratory to test various ad strategies and to make adjustments in real time based on the results [32]. With online ads, it is possible to track a number of “denominators,” including the number of times an ad is viewed, the number of ad clicks, the number of individuals who register for cessation treatment in response to an online ad, and the cost of recruiting smokers to treatment. This real-time evaluation of consumer demand permits continuous quality improvement throughout a marketing initiative so that if an ad is not performing well in terms of recruitment volume or cost-effectiveness, it can literally be replaced within hours.

Online advertising is extremely sophisticated, with billions of dollars spent by marketing agencies each year [33,34]. However, the science of how to develop and test online advertising as a mechanism to boost consumer demand for behavioral health interventions is in its infancy [19]. Several studies in HIV and sexually transmitted disease (STD) prevention research have used online advertisements to recruit participants [35-39], but details about their effectiveness and cost-efficiency is limited. Smoking-related studies have used online advertising to recruit smokers to Web-based cessation programs [40,41] but provide little information about the approaches used. Other cessation studies have incorporated the Internet into recruitment efforts but have not used banner or paid search ads [42-45]. We know of no published studies that have examined the cost-effectiveness of online advertising or compared online advertising to traditional media approaches in recruiting smokers to cessation programs.

The aims of this study are (1) to demonstrate the feasibility of online advertising as a strategy to increase consumer demand for cessation treatments, (2) to illustrate the tools that can be used to track and evaluate its impact on consumers by means of a comparison between traditional media campaigns and online marketing campaigns, and (3) to highlight some of the methodological challenges and future directions for researchers who wish to advance the science of online advertising. This study represents a preliminary investigation into the use of online advertising to cost-efficiently reach, recruit, and engage smokers in cessation treatments, and it raises a number of important questions regarding methodological issues unique to this type of research.

## Methods

### Overview of Settings and Advertising Campaigns

The study was conducted between December 1, 2004 and October 31, 2006 as a partnership between Healthways QuitNet LLC, ClearWay Minnesota, and the New Jersey Department of Health. During the study period, online advertising campaigns were run to promote QuitNet’s Web-based cessation program [13] and the state-run telephone quitlines in Minnesota and New Jersey. The advertising campaigns were managed by Healthways QuitNet, including negotiation of contracts with online advertising partners.

### Procedure

This feasibility study used an observational design consisting of the delivery of advertising campaigns within two conditions: an online condition and a traditional mass media (comparison) condition. In both conditions, the timing of the advertising campaigns, delivered in two states, permitted their impact to be evaluated on several dependent variables: (1) number of individuals who enrolled in a cessation treatment, (2) the demographic, smoking, and treatment utilization characteristics of smokers recruited to treatment, and (3) the cost to enroll a smoker in treatment.

#### Online Advertising Condition

Within the online condition, several creative approaches and advertising partners were developed.

##### Creative Approaches

All online ads were banner or paid search (text) ads. There were four categories of creative approaches used in ads during the study period (see Multimedia Appendix for samples):

1. Ads that focused on the importance of getting support during the quitting process (eg, “Don’t quit alone, Quit with us,” “Remember the buddy system? It works for quitting too”);
2. Ads that used humor to capture the attention of Internet users (eg, “Cold turkey is good for sandwiches, not for quitting”);
3. Ads that used website-specific concepts (eg, “Quitting has its highs and lows, but favorable conditions are on the way” placed on Weather.com)
4. Paid search (text) ads (eg, “New Jersey QuitNet: Expert support, free resources, & med advice”)

##### Advertising Partners

There were three categories of advertising partners: (1) national websites, (2) local websites, and (3) search engines. Banner ads were placed on national and local websites and were purchased on a per impression basis using a negotiated cost per mille (CPM) rate, the cost of showing the ad to one thousand viewers. National websites included Yahoo!, AOL, Weather.com, 24/7 Real Media, and WebMD. All five sites were selected as advertising partners based on their broad reach. In addition, WebMD was selected to target Web users seeking health information. Local websites in Minnesota were StarTribune.com and PostBulletin.com, and NJ.com was used in New Jersey.

Paid search ads were run on search engines and were purchased on a per click basis. Two types of campaigns were run on each search engine: (1) Geo-targeted campaigns displayed ads to Internet users in a specific geographic region based on their IP address. Generic keywords such as “quit smoking” or “stop smoking” can be used in this type of campaign. (2) Country-wide campaigns displayed ads to Internet users throughout the United States. In order to reach the target populations in Minnesota and New Jersey, ads included keywords such as “NJ Quit Smoking.” The search engines used in this study included Google, MSN Adcenter, and Overture (now part of Yahoo! Search). These search engines are known to have a user base with slightly different demographic characteristics [46], which provides additional opportunities for targeting specific segments of smokers. Banner ads were displayed prominently on the side or top of advertising partner websites, and paid search ads were displayed on search engine results pages.

##### User Experience

All ads included a call to action that instructed the viewer to “click here” to get more information. Clicking on the ad took the user to a landing page where he or she read a brief description of three cessation treatment options: (1) 24/7 online support, (2) telephone counseling, or (3) telephone and online support. If users selected the first option, they were taken immediately to the state-sponsored QuitNet website where they were prompted to register and begin using the website. If the individuals selected the second or third option, they were asked to fill out an online quitline referral form, which provided a quitline counselor with basic contact information; individuals selecting option three were then directed to the state-sponsored QuitNet website to register.

#### Traditional Media Comparison Condition

At the same time as the online advertising campaigns, a number of traditional media campaigns were also run in Minnesota and New Jersey to promote the QuitNet website. These campaigns included billboards, television and radio ads, outdoor advertising (eg, bus sides, bus shelters), direct mail, and physician detailing. To ensure a consistent look and feel between the traditional media campaigns and online ads, the same creative approaches were used. In the majority of cases, identical content was used for traditional media and online ads; however, in some cases, the similarity in creative approach focused primarily on the use of the same logo and color scheme. Only the traditional media ads listed the QuitNet URL in the call to action since this information was not necessary in online ads.

Free search engine listings also generate a high volume of traffic to the QuitNet website [47]. As a result of this heterogeneous mix of marketing and promotion efforts, a large number of smokers register to use QuitNet’s service each month [47]. As a comparison group, we extracted registration data on all individuals who joined QuitNet during the study period in response to all other forms of advertising (ie, not an online ad).

### Measures

#### Online Ad Tracking Data

When an Internet user clicked on an online ad, a unique event identifier was stored on the Healthways QuitNet server along with information about the specific ad that had been displayed, the website on which it was displayed, and the type of cessation program that the individual chose from the landing page (Web only, phone only, Web plus phone, no action).

#### Phone and Web Registration Data

The unique identifier was also linked to registration data if the individual registered with QuitNet and/or telephone counseling. The QuitNet registration process asked individuals to select a username and password and to indicate their age, gender, race, education level, smoking rate, time to first cigarette, readiness to quit smoking, and reason for registration. Registration data for telephone counseling included email address, first and last name, telephone number, preferred contact time, and consent to share this information with a quitline counselor.

#### QuitNet Utilization Data

The following utilization metrics were extracted from the QuitNet database for all registered QuitNet users: number of log-ins, minutes per log-in, number of page views, and interactions with other users and expert counselors. Site usage was tracked by the system through short-term (length of session) and long-term (persistent between sessions) cookies, allowing for identification of users throughout their life cycle whether logged in or not. Utilization data for telephone counseling were gathered by the individual vendors in Minnesota and New Jersey and were not available at the time of this analysis.

### Statistical Analyses

We summarize data regarding response rates, treatment enrollment, and costs using relative frequencies for categorical variables and descriptive statistics for continuous variables. Univariate analyses were conducted to examine differences on demographic and smoking variables between traditional media and online ad responders. Statistical significance levels were calculated using *t* tests to examine mean differences in continuous variables and chi-square or nonparametric tests to examine differences in proportions of categorical variables. For categorical variables with more than two levels, squared Pearson residuals were investigated to examine the source of any statistically significant differences.

## Results

### Overall Response Rates

**Figure 1:**
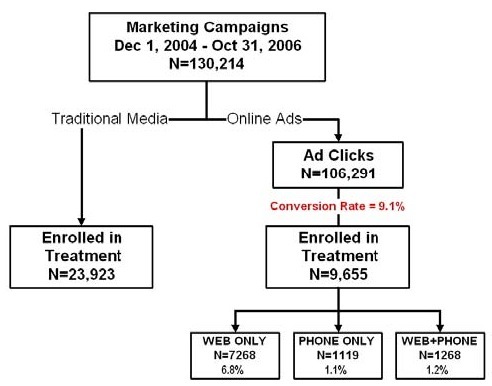
Response rates for online and offline ads

As shown in Figure 1, a total of 130,214 unique identifiers were created on the Healthways QuitNet server during the study period: 23,923 (18.4%) were for traditional media responders who registered with QuitNet, and 106,291 (81.6%) were for online ad clicks. Of the online ad clicks, 9655 individuals (9.1%) registered for some form of cessation treatment: 6.8% (n = 7268) selected Web only, 1.1% (n = 1119) selected phone only, and 1.2% (n = 1268) selected both Web and phone.

### Characteristics of Users Recruited via Online Ads Versus Traditional Media

We examined differences in demographic and smoking variables between treatment users recruited via online ads and those recruited via traditional media. As shown in Table 1, there were differences for all variables examined. Compared to traditional media, online ads recruited a higher percentage of 18- to 24-year-olds (*P* < .001), men (*P* < .001), non-White individuals (*P* < .001), those with a high school degree or less (*P* = .02), those who had not yet quit smoking (ie, those in precontemplation, contemplation, or preparation phase of motivational readiness, *P* < .001), and those who smoked within 30 minutes of waking (*P* < .001). In addition, online ad responders smoked more (*P* = .002), although the very small mean difference (19.0 cigarettes/day vs 18.7 cigarettes/day) is not likely to be clinically meaningful.

**Table 1:** Online vs traditional recruitment: demographic and smoking characteristics ^a^

	Online Recruitment (n = 8536) ^b^	Traditional Recruitment (n = 23,923)	*P*
**Age (years)**			< .001
18-24	14.1	12.6	
25-44	57.3	59.2	
45-64	27.5	26.8	
65+	1.1	1.4	
Male	41.7	38.3	< .001
**Race/Ethnicity**			< .001
White	83.9	88.2	
Black / African American	5.5	3.9	
Hispanic	4.3	3.1	
Asian / Pacific Islander	3.5	2.5	
Native American / Aboriginal	0.6	0.7	
Other	2.2	1.6	
**Education**			.02
High school / GED ^c^ or less	24.6	23.2	
Some college / junior college	40.9	40.2	
College grad or higher	34.5	36.6	
**Motivational readiness**			< .001
Never smoker	0.8	1.7	
Precontemplation	1.5	1.0	
Contemplation	40.4	36.5	
Preparation	45.7	42.6	
Action	10.7	16.5	
Maintenance	0.9	1.7	
Cigarettes per day, mean (SD)	19.0 (9.7)	18.7 (9.4)	.002
**Time to first cigarette**			< .001
Less than 30 minutes	72.1	69.2	
30 minutes or more	27.9	30.8	

^a^ Values are percentages unless otherwise noted.

^b^ Does not include the 1119 individuals who registered for “phone only” since demographic data are not available on this group.

^c^ General equivalency diploma.

Within the online ad condition, we examined differences between those who responded to paid search ads (n = 6163) and those who responded to banner ads (n = 2271). The rationale for these analyses was to explore whether individuals actively searching for cessation assistance via search engines (paid search responders) are different from those who respond to a more “passive” advertising strategy (banner ads). There were significant differences across the demographic categories we examined, with banner ads recruiting a larger percentage of 18- to 24-year-olds (14.9% vs 13.8%, *P* < .001), men (43.7% vs 41.0%, *P* = .03), non-White individuals (20.7% vs 14.6%, *P* < .001), those with a high school education or less (26.3% vs 23.9%, *P* = .03), and those who had not yet quit smoking (92.7% vs 82.7%, *P* < .001). Banner ad responders smoked slightly less than paid search responders (mean = 18.6, SD = 9.9 vs mean = 19.2, SD = 9.6; *P* = .01), although this statistically difference is not likely to be clinically meaningful. There were no differences between paid search responders and banner ad responders on time to first cigarette.

We also compared online and traditional media responders on website utilization metrics to determine if online ad responders engaged with the QuitNet website to the same degree. The metrics examined included the total number of log-ins during the study period, average number of minutes per session, total time spent online, total number of pages viewed, and three metrics of community participation: the percentage who posted in one of the community forums, the percentage who sent email through QuitNet’s internal messaging system (QMail) to at least one other person, and the percentage who received QMail from at least one other person. Consistent with other studies [13,40], patterns of website utilization varied widely; therefore, given the very large skewness of these utilization variables, we report the median and interquartile range in Table 2. There were statistically significant differences on all metrics examined, with higher website utilization among traditional media responders. Although statistically significant due to the enormous power in this study, differences in website utilization are small in magnitude and not likely to be clinically meaningful.

**Table 2:** Median (interquartile range) of QuitNet utilization among online and traditional media responders ^a^

	Online Recruitment (n = 8536)	Traditional Recruitment (n = 23,923)	*P*
Number of log-ins	1 (1-3)	1 (1-4)	< .001
Average session length (minutes)	12 (8-19)	12 (8-20)	.01
Total minutes spent online	20 (10-47)	24 (12-63)	< .001
Total pages viewed	35 (20-76)	40 (22-98)	< .001
Percentage posting at least once in public forums	8.3	11	< .001
Percentage who sent Qmail to at least 1 person	28.1	35.5	< .001
Percentage who received Qmail from at least 1 person	6.4	10	< .001

^a^Between-group differences were analyzed using Wilcoxon W test for continuous data (median) and chi-square (proportions).

### Reach to Subgroups of Smokers by Selected Creative Approaches

Next we explored whether certain online ads were more effective in recruiting specific subgroups of smokers than traditional media. As shown in Table 3, we examined the percentage of individuals in specific demographic and smoking subgroups recruited by traditional media approaches and compared it to the percentage recruited by specific types of online ads (ie, quitting with support, humorous, text ads). We focused on subgroups of smokers that are traditionally underrepresented in most cessation programs, including males, younger adults, racial/ethnic minorities, and those with lower levels of education. These analyses examined the impact of creative approach across multiple websites on which ads were placed in order to control for the potential influence of variations in website demographics [46]. For this reason, we excluded the creative approach that used website-specific concepts since ads were only placed on two websites (WebMD and Weather.com).

Compared to traditional media, humorous ads were effective in recruiting a higher percentage of males (*P* < .001), young adults (*P* < .001), non-White individuals (*P* < .001), and those with a high school degree or less (*P* = .004). Humorous ads were also the only creative approach that was effective in recruiting smokers with lower levels of education. Ads that focused on the importance of support during quitting recruited a higher percentage of young adults (*P* < .001) and non-White individuals (*P* < .001) compared to traditional media. Paid search ads recruited a higher percentage of males (*P* < .001), young adults (*P* = .004), and non-White individuals (*P* < .001).

**Table 3:** Demographic characteristics of individuals recruited to cessation treatment by recruitment approach

Recruitment Approach	Male (%)	Age 18-24 Years (%)	Non-White (%)	High School Degree or less (%)
Traditional media (comparison group)	38.2	12.5	11.8	23.2
Humor (online)	44.1 ^a^	15.9 ^a^	20.6 ^a^	26.8 ^b^
Quit with support (online)	39.8	13.4 ^a^	21.2 ^a^	26.4
Paid search (online text ad)	41.0 ^a^	13.8 ^b^	14.6 ^a^	23.9

^a^ *P* < .001 (compared to traditional media).

^b^ *P* < .01 (compared to traditional media).

### Cost-Effectiveness

To illustrate how the real-time data collection of online ads can be used to rapidly inform impact and improve cost-effectiveness, we present the results of selected online advertising campaigns run in New Jersey during two specific time periods: December 2004 to May 2005 and August 2006 to October 2006. During the entire study period, several ads were tested across multiple websites, each with cost-effectiveness results. The findings presented in Table 4 and Table 5 from these selected campaigns are designed to highlight the types of metrics that can be analyzed and several of the most important lessons learned.

It is important to note that online media campaigns typically evaluate effectiveness by the click-through rate and the average cost per click. The click-through rate is a ratio of the number of clicks divided by the number of impressions, and the average cost per click is the fee charged by an advertiser divided by the number of clicks on an ad. While both metrics are useful measures of the performance of an individual ad and allow for comparison of ads across websites, they do not reflect the actual costs of getting an individual smoker *enrolled* in a smoking cessation treatment program. Thus, in the tables below, we present the total cost of each campaign, the number of individuals who clicked on ads during the campaign, and the number that enrolled in a cessation treatment program. The conversion rate is a ratio of the number of individuals who registered with a cessation program (Web only, phone only, Web plus phone) divided by the total number of individuals who clicked on the ads. The cost per registrant is calculated by dividing the total amount spent in a campaign by the number of cessation program registrants.

Table 4 shows the results of a series of campaigns that were run between December 2004 and June 2005. The WebMD campaign was stopped after 1 month due to the low conversion rate and extremely high cost per registrant, and funds were allocated to a subsequent campaign with Google (Phase II). Similarly, funds were reallocated from the NJ.com campaign to Google (Phase II) in June 2005 given the low yield of ads on NJ.com.

With a total budget of US $45,000, the online ads run on these six advertising sites during a 6-month period resulted in a total of 1285 individuals who registered with a cessation program. The average conversion rate was 9%, with a range of 2% to 16%. The only advertising partner to yield a conversion rate higher than 10% was Google, with the other partner sites producing much lower conversion rates. The average cost per registrant was US $35, with a range of US $7-$476.

**Table 4:** Cost-effectiveness in early New Jersey campaigns (December 2004 to June 2005)

Advertiser	Spend (US $)	Number of Ad Clicks	Number Registered for Cessation Treatment	Conversion Rate	Cost per Registrant (US $)
Google Phase I	$5000	4651	762	16%	$7
Yahoo	$10,000	3769	265	7%	$38
AOL	$5000	1390	44	3%	$114
Weather.com	$10,000	1238	92	7%	$109
WebMD (Feb 1-28)	$10,000	476	21	4%	$476
NJ.com (Jan 1-May 31)	$4530	2061	39	2%	$116
Google Phase II (June 1-31)	$470	546	62	11%	$8
**Total Media Spend**	**$45,000**	**14,131**	**1285**	**9%**	**$35**

Using lessons learned from these campaigns and others, a different series of ads were run in New Jersey between August 2006 and October 2006, as shown in Table 5. During this 3-month period, 751 individuals registered with a cessation program at an average cost of US $38 per registrant. Although the overall conversion rate was slightly lower than in the earlier series of campaigns, three advertising partners produced conversion rates higher than 10%. In addition, the cost per registrant was far lower across advertising partners, with three of the sites averaging less than US $20 per registrant. In addition, ad placement appears to have improved based on the overall number of ad clicks (n = 11,110) during this 3-month time period as compared to 14,131 ad clicks during the previous 6-month campaign.

**Table 5:** Cost-effectiveness in later New Jersey campaigns (August 2006 to October 2006)

Advertiser	Spend (US $)	Number of Ad Clicks	Number Registered for Cessation Treatment	Conversion Rate	Cost per Registrant (US $)
Overture	$109	115	24	21%	$5
MSN	$112	88	18	20%	$6
Google	$7651	4143	476	11%	$16
Yahoo	$10,011	2008	135	7%	$74
AOL	$4999	2478	71	3%	$70
24/7 Media	$6000	2163	27	1%	$222
**Total Media Spend**	**$28,882**	**11,110**	**751**	**7%**	**$38**

## Discussion

To reduce the population prevalence of smoking, innovative marketing initiatives are needed to increase awareness and utilization of proven cessation treatments. Data from this preliminary study are among the first to demonstrate the feasibility of using online advertising to recruit smokers to Web- and telephone-based cessation programs. Online advertising in just two states resulted in cessation treatment utilization by over 9600 smokers, almost half the number of registered users during the same time period as all other forms of advertising throughout the United States. If online advertising were implemented on a national basis, it could potentially result in more than 200,000 smokers using Web- or telephone-based cessation treatment.

Since the results of these analyses were based on an observational study within the context of a set of real-world interventions, the assumptions made in interpreting the results and the inability to control for or rule out potential confounders implies that the conclusions are tentative and subject to alternative explanations. They are used to illustrate the feasibility and potential of online advertising, as well as some of the challenges and caveats that researchers will need to consider in terms of design, sampling, methods, and measurement in this new area of research. This paper explores new territory, and, since it was not designed at the outset as a research study, the discussion that follows should be considered as illustrative of the potential for this new informatics and communications technology to strengthen the science of dissemination as well as the dissemination of the science [48].

These results illustrate the ability of online ads to reach specific subgroups of smokers that may not traditionally seek cessation assistance [49,50]. Compared to traditional media, online ads recruited a higher percentage of males, young adults, racial/ethnic minorities, and those with a high school degree or less, with banner ads driving much of the effect. Smoking continues to be more prevalent among these groups [51], making it critical to identify effective methods to increase consumer demand for cessation treatments. The potential of online advertising to reach and recruit smokers from these subgroups is promising. Website utilization data suggest that online ad responders are not merely “casual browsers” who happened to click on an online ad, but rather smokers who engaged with the website to a similar extent as smokers recruited via other channels. It is also interesting to note that online ads attracted a small percentage of “never smokers” despite the fact that messages targeted current smokers. Recently, research has focused on interventions designed for support persons as a way of increasing the reach of cessation treatments to smokers themselves [52]. Online advertising could be used to recruit support persons for these types of interventions.

We also examined the effectiveness of various creative approaches in recruiting specific types of smokers. Humorous ads were the most effective in reaching subgroups of smokers who are less likely to participate in cessation treatment and were the only creative approach that recruited a higher percentage of those with a high school degree or less compared with traditional media. It is interesting to note that ads focused on the importance of getting support did not increase the percentage of men using cessation treatment over traditional media, whereas paid search ads did recruit a higher percentage of men. These data provide just an initial glimpse into the many types of analyses that are possible and also raise important questions that should be addressed in future research where rigorous, controlled research designs could be employed to tease apart specific parametric effects. Specifically, what types of communication messages or creative approaches are most effective for specific subgroups of smokers? Are gain-framed or loss-framed messages more effective in online advertising in recruiting smokers to treatment? To what extent is cultural targeting important, and is targeting at the surface level (ie, matching of materials and messages to characteristics of the target population) or deep level (ie, messages and interventions incorporate information about cultural, social, environmental, psychological, and historical factors that differentially influence health behaviors) more effective?

This study is also the first to present detailed information about the cost-effectiveness of various online advertising strategies. Consistent with a social implementation approach to cost-effectiveness, we excluded costs associated with development and preparation [53]. On average, it cost US $36 to recruit a smoker to Web- or phone-based treatment using online ads. Paid search advertising on search engines tended to be the most cost-effective approach, with a cost of US $5-$8 per registrant. Paid search also yielded the highest absolute number of registrants and the highest conversion rates. These results compare favorably to the few reports of costs associated with traditional recruitment methods, which range from US $19-$500 per enrolled smoker depending on the recruitment channel [54-57]. The ability to closely monitor the performance of ads resulted in pulling funds from two underperforming campaigns (WebMD and NJ.com) and reallocating these funds to a campaign that more than doubled the conversion rate and dramatically reduced the cost per registrant.

This study employed an observational approach to determine preliminary feasibility and cost-effectiveness of online advertising. Future research studies will need to identify the most rigorous and appropriate research designs and statistical methods to be used with this kind of “real-world” data. In particular, the balance between internal and external validity must be carefully considered [58]. Many of the threats to internal validity inherent in this kind of research can be addressed by giving careful consideration to research design and methodological issues, while at the same time strengthening the external validity of the study by, for example, specifying the characteristics and behavior of users at each level of “denominator” in a manner that is simply not possible with traditional media campaigns. Studies of traditional mass media often use interrupted time series designs with a comparison group. Geo-targeting in Internet research may permit the same type of comparison group to be created. Time series regression analysis may be the most appropriate statistical approach for interrupted time series designs [19] as it accounts for underlying time trends and auto-correlation between individual observations. Quasi-experimental Latin Square designs [59] and adaptive clinical designs as discussed by Collins et al [60] may also be ideally suited for this type of research. Future research should also explore the degree to which various online advertising strategies are linked to treatment utilization and/or cessation outcomes.

Results should be considered within the context of several other limitations. First, a percentage of those who completed the quitline referral form may have been unreachable or uninterested in telephone counseling, so the estimates of those recruited to “phone only” may have be inflated. Second, this study relied on cookies to track website utilization among registered QuitNet members. Individuals who regularly delete cookies from their computer cannot be tracked with the same accuracy as those who do not. Related to this issue, cookies (along with email address) were also used to minimize duplicate enrollment on QuitNet. It is possible that the same individual could be represented more than once as a QuitNet user if they had deleted cookies, registered from a different computer, and/or registered using a different email address. It is also possible that some individuals were represented more than once in the total number of online ad clicks (n = 106,291) as it was not possible to identify unique individuals in this process. Third, a small percentage (< 3%) of those who registered on the QuitNet website indicated that they were seeking assistance for someone else rather than for themselves. For this preliminary study, we elected to include them in the total number of individuals who registered for some type of treatment given the growing literature on the importance of support persons. Future studies may consider analyzing this subgroup separately. Finally, it is possible that online ad responders had seen promotional materials in other (offline) places prior to clicking on a banner ad or paid search ad. This prior exposure may have primed them to click on an online ad. It will be important for future research studies to more clearly understand this “multiple exposure” effect.

In summary, multiple marketing and promotion channels are critical to raise awareness of the importance of cessation, to motivate smokers to consider cessation, and to link smokers to proven cessation treatments [18]. Online advertising is forecast to experience enormous growth over the next 3 to 5 years, encroaching on budgets typically allocated for traditional media [33,34]. This feasibility study illustrates the potential of online advertising to promote the utilization of evidence-based cessation treatment. The tools available to track and evaluate program, process, and outcome metrics require careful consideration of both internal and external validity issues in designing research studies in this new domain. The technology and tools available can both strengthen the science of dissemination and also help improve interventions that will more effectively disseminate the products of evidence-based science. Despite the limitations of this observational study, the results provide useful and informative preliminary support to warrant more investment in the design and conduct of future studies to advance the science of marketing and consumer demand in the Internet age.

## Multimedia Appendix 1

Examples of online ads (focused on support, humor, website-specific concepts, and paid search (text) ads). Note: Some ads are animated, but the animation will not show in the PDF file. For the original gif files download Multimedia Appendix 2.

## Multimedia Appendix 2

Examples of online ads, original jpg/gif files (compressed). To view animated gif's, open files with your web browser (eg. Internet Explorer) or similar software.
