# Correction: Online Advertising as a Public Health and Recruitment Tool: Comparison of Different Media Campaigns to Increase Demand for Smoking Cessation Interventions

**DOI:** 10.2196/jmir.1212

**Published:** 2009-01-16

**Authors:** Amanda L Graham, Pat Milner, Jessie E Saul, Lillian Pfaff

**Affiliations:** ^4^New Jersey Department of Health and Senior ServicesTrentonNJUSA; ^3^ClearWay MinnesotaMinneapolisMNUSA; ^2^Healthways QuitNet LLCBostonMAUSA; ^1^Georgetown University Medical Center / Lombardi Comprehensive Cancer CenterWashingtonDCWashingtonDCUSA

A number of errors regarding the cited references occurred in the article by Amanda Graham et al. (J Med Internet Res 2008;10(5):e50). The corrected version was republished on the JMIR website on 14.01.2009 at http://www.jmir.org/2008/5/e50 and resubmitted to PubMed Central; however, we have no control over other websites and aggregators which may mirror content from JMIR and may not update the original version. The corrected version can be identified by citing 60 references, while the originally published version cited only 57 references. Beginning with reference #13, citations in the text were mismatched with references at the end of the manuscript. In addition, the authors inadvertently omitted three references (#48, #58, and #59 in the corrected version).

