# Supplementary material for: Online Advertising as a Public Health and Recruitment Tool: Comparison of Different Media Campaigns to Increase Demand for Smoking Cessation Interventions
Source: J Med Internet Res. 2008 Dec 15;10(5):e50. doi: 10.2196/jmir.1001 (PMC2630839; doi:10.2196/jmir.1001)

Multimedia Appendix 1

Cite as:
Graham AL, Milner P, Saul JE, Pfaff L
Online Advertising as a Public Health Tool: Comparison of Different Recruitment Campaigns for Smoking Cessation Interventions
J Med Internet Res 2008;10(5):e50
<URL: http://www.jmir.org/2008/5/e50/>

Examples of online ads focused on support

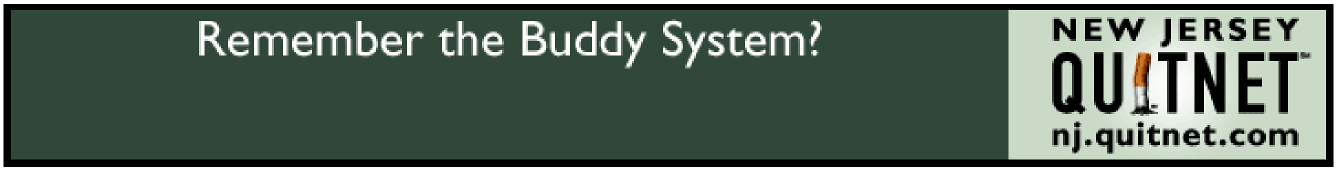

(animated gif file – animation will not show in the PDF file – please download Appendix 2 for the original gif file)

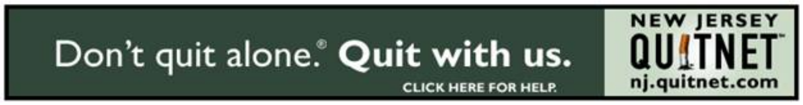

Examples of online ads focused on humor

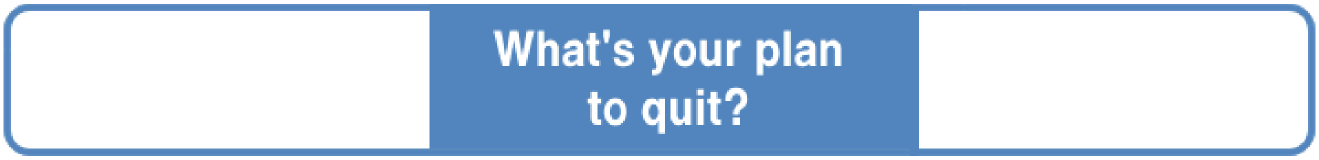

(animated gif file – animation will not show in the PDF file – please download Appendix 2 for the original gif file)

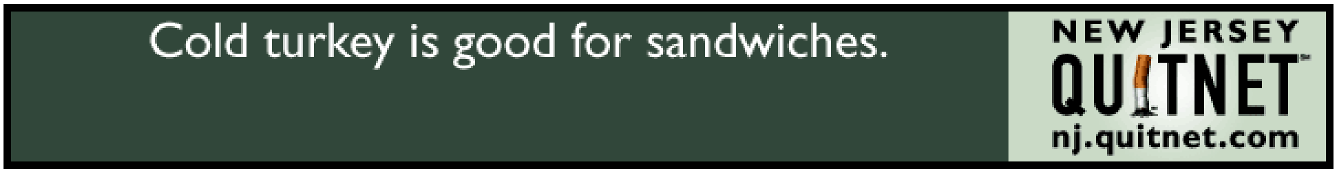

Examples of online ads using website-specific concepts (e.g. weather.com)

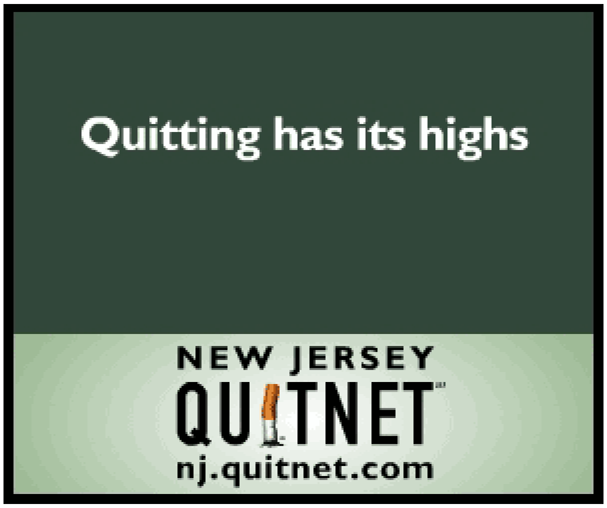

(animated gif file – animation will not show in the PDF file – please download Appendix 2 for the original gif file)

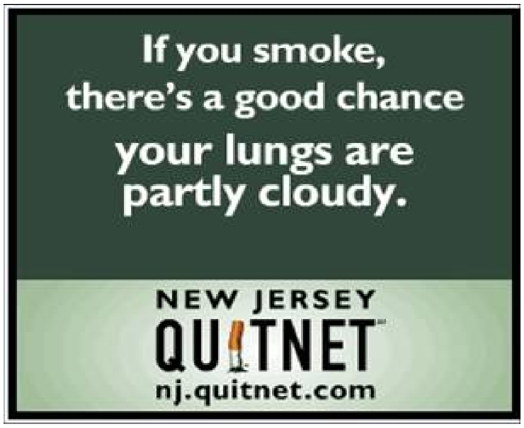

Examples of paid search (text) ads

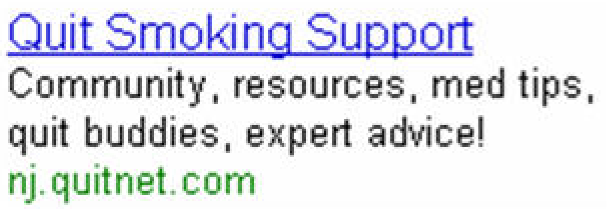

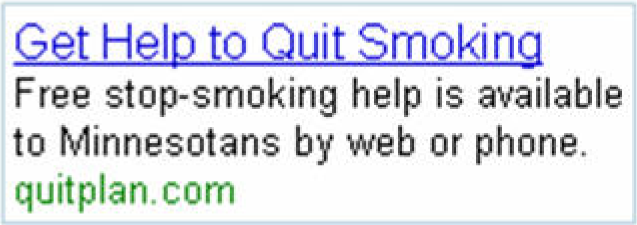

Supplement: Supplementary file 1 [file jmir_v10i5e50_app1.pdf]
