# Supplementary figures and images for: Online Advertising as a Public Health and Recruitment Tool: Comparison of Different Media Campaigns to Increase Demand for Smoking Cessation Interventions
Source: J Med Internet Res. 2008 Dec 15;10(5):e50. doi: 10.2196/jmir.1001 (PMC2630839; doi:10.2196/jmir.1001)

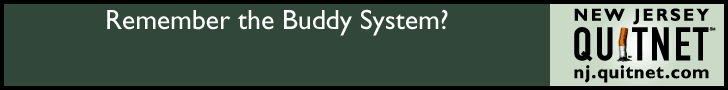

Supplement: Supplementary file 2 [file jmir_v10i5e50_app2.zip › 1001-3608-2-SP.gif]

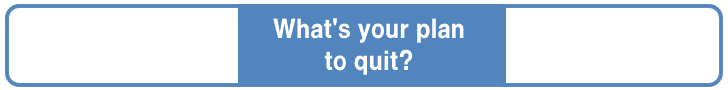

Supplement: Supplementary file 2 [file jmir_v10i5e50_app2.zip › 1001-5104-2-SP.gif]

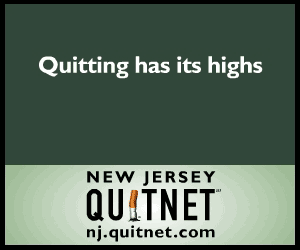

Supplement: Supplementary file 2 [file jmir_v10i5e50_app2.zip › 1001-5105-1-SP.gif]

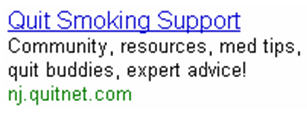

Supplement: Supplementary file 2 [file jmir_v10i5e50_app2.zip › 1001-5106-1-SP.jpg]

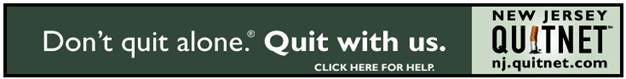

Supplement: Supplementary file 2 [file jmir_v10i5e50_app2.zip › 1001-5107-1-SP.jpg]

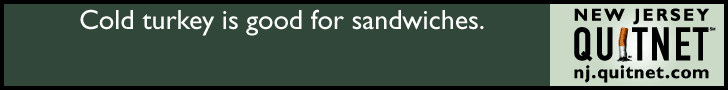

Supplement: Supplementary file 2 [file jmir_v10i5e50_app2.zip › 1001-5108-1-SP.gif]

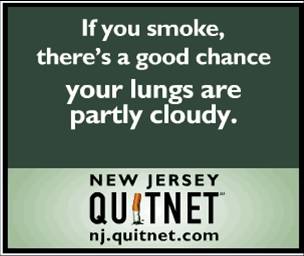

Supplement: Supplementary file 2 [file jmir_v10i5e50_app2.zip › 1001-5109-1-SP.jpg]

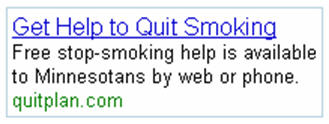

Supplement: Supplementary file 2 [file jmir_v10i5e50_app2.zip › 1001-5110-1-SP.jpg]
